# Supplementary material for: A Knowledge-Graph-Based Multimodal Deep Learning Framework for Identifying Drug–Drug Interactions
Source: Molecules. 2023 Feb 3;28(3):1490. doi: 10.3390/molecules28031490 (PMC9919258; doi:10.3390/molecules28031490)
Supplement: Supplementary file 1 [file molecules-28-01490-s001.zip › Supplementary Information S1.pdf]

Supplementary Information S1：

Detailed steps for data collection and knowledge graph construction

Step 1. Download drugs and their structure information of smiles from the DrugBank database (Version 5.1.8), and get 11168 drug-chemical structure pairs.

Step 2. The Morgan molecular fingerprint descriptor (MorganFP, 1024 dimensions) of each drug were calculated using RDKit software, delete 9 drugs that can not calculate Morgan molecular fingerprint descriptor, and obtain a drug matrix of size 11159*1024.

Step 3. Download the corresponding drug-drug interactions, targets and enzymes of 11150 drugs from the DrugBank database. Obtain 1113431 drug-drug interactions (drugs:3360) after removing duplicates, 15190 drug-target associations after removing the non-human proteins (drugs:5118, targets:2725), 5085 drug-enzyme associations (drugs:1577, enzymes:353) after removing the non-human proteins.

Step 4. Download CTD_chemicals_genes, CTD_chemicals_diseases and CTD_chemicals_diseases file from CTD database. For CTD_chemicals_genes, non-human genes were deleted, the name, synonymous name or CAS number of the chemical were used to match the drug in DrugBank, and if the match is successful, the association pair is collected. Obtain 9310 drug-gene associations (drugs:427, genes:8083). For CTD_chemicals_diseases and CTD_chemicals_diseases, removed drug-disease pairs without annotation "therapeutic" in the field of Direct Evidence, meaning that the obtained drugs have therapeutic effects on diseases, obtained 64879 drug-disease associations (drugs:2902, disease:3065), and 16634 gene-disease associations (genes:3466, disease:3551).

Step 5. Download protein-protein interaction pairs (PPIs) from the HIPPIE database, self-interactions, repeat interactions, and proteins without Uniprot id and/or sequence information were deleted, those without targets or enzymes in PPIs were deleted, and get 150338 PPIs (including 15005 proteins).

Step 6. Download protein-disease associations from the UniProt database, irrelevant association pairs were deleted, and get 6722 protein-disease associations (protein:3679, disease:1347).

Step 7. Integrate all data to construct the knowledge graph so that the collected drugs meet these conditions: the Morgan molecular fingerprints can be calculated by smiles, and at least one of genes, protein and diseases exists. Finally, the date was obtained 35975 entities including 7129 drugs, 8083 genes, 15184 protein, and 5579 diseases. And the indexes of these entities were built as input to model KGCN.

Table S1 Details of the knowledge graph.

Type of nodes	Count	Relations	Count	Source
Drug	7129	Drug-Drug	1113431	DrugBank
Gene	8083	Drug-Target	15190	DrugBank
Protein	15184	Drug-Enzyme	5085	DrugBank
Disease	5579	Drug-Gene	9310	CTD
		Drug-Disease	64879	CTD
		Gene-Disease	16634	CTD
		Protein-Protein	150338	HIPPIE
		Protein-Disease	6722	UniProt
Total	35975	Total	1381589	

Table S2 The impact of different aggregators on model performance (mean ± RSD(%)).

Type of aggregator	Acc(%)	Sen(%)	Spe(%)	Pre(%)	F1	MCC	AUC	AUPR
Sum	98.61 ± 0.02	98.74 ± 0.09	98.48 ± 0.11	98.49 ± 0.11	0.9862 ± 0.02	0.9723 ± 0.05	0.9992 ± 0.01	0.9992 ± 0.01
Neigh	98.67 ± 0.02	98.76 ± 0.01	98.58 ± 0.05	98.58 ± 0.05	0.9867 ± 0.02	0.9734 ± 0.05	0.9992 ± 0.01	0.9993 ± 0.01
Concat	98.64 ± 0.04	98.65 ± 0.12	98.64 ± 0.09	98.64 ± 0.09	0.9864 ± 0.04	0.9729 ± 0.08	0.9992 ± 0.01	0.9992 ± 0.01
